# Molecular Dynamics Study of the Opening Mechanism for DNA Polymerase I

**DOI:** 10.1371/journal.pcbi.1003961

**Published:** 2014-12-04

**Authors:** Bill R. Miller, Carol A. Parish, Eugene Y. Wu

**Affiliations:** 1 Department of Biology, University of Richmond, Richmond, Virginia, United States of America; 2 Department of Chemistry, University of Richmond, Richmond, Virginia, United States of America; Max Planck Institute for Biophysical Chemistry, Germany

## Abstract

During DNA replication, DNA polymerases follow an induced fit mechanism in order to rapidly distinguish between correct and incorrect dNTP substrates. The dynamics of this process are crucial to the overall effectiveness of catalysis. Although X-ray crystal structures of DNA polymerase I with substrate dNTPs have revealed key structural states along the catalytic pathway, solution fluorescence studies indicate that those key states are populated in the absence of substrate. Herein, we report the first atomistic simulations showing the conformational changes between the closed, open, and ajar conformations of DNA polymerase I in the binary (enzyme∶DNA) state to better understand its dynamics. We have applied long time-scale, unbiased molecular dynamics to investigate the opening process of the fingers domain in the absence of substrate for *B. stearothermophilis* DNA polymerase *in silico*. These simulations are biologically and/or physiologically relevant as they shed light on the transitions between states in this important enzyme. All closed and ajar simulations successfully transitioned into the fully open conformation, which is known to be the dominant binary enzyme-DNA conformation from solution and crystallographic studies. Furthermore, we have detailed the key stages in the opening process starting from the open and ajar crystal structures, including the observation of a previously unknown key intermediate structure. Four backbone dihedrals were identified as important during the opening process, and their movements provide insight into the recognition of dNTP substrate molecules by the polymerase binary state. In addition to revealing the opening mechanism, this study also demonstrates our ability to study biological events of DNA polymerase using current computational methods without biasing the dynamics.

## Author Summary

All organisms are dependent on the proper replication of their DNA for survival. DNA polymerase is the enzyme responsible for copying our DNA during cell division. We have performed computational simulations on DNA polymerase to understand the fundamental dynamics of the enzyme. Our simulations provide new information about the way polymerase moves in solution that is not obtainable through traditional experimental techniques. In particular, we investigated the dynamics of DNA polymerase “opening” in the binary state (enzyme+DNA with no nucleotide substrate) starting from three different conformations. The results are consistent with available experimental data on the relative conformations of DNA polymerase in the binary state. Furthermore, we identified a novel intermediate species that we hypothesize plays a role in the dynamics of nucleotide substrate binding. Additionally, we determined the previously unknown ordering of events in the opening mechanism, and suggest new details about how the polymerase may interact with an incoming nucleotide substrate. Lastly, this research serves as a proof of principle that we can use our methodology to perform long-time scale computational simulations on DNA polymerase to explain currently unknown phenomena surrounding DNA replication.

## Introduction

DNA polymerase, which is responsible for copying DNA, is a vital enzyme involved in the transfer of genetic information for living organisms. It is also utilized by scientists to replicate DNA sequences during polymerase chain reactions (PCR). DNA polymerase has the ability to quickly and accurately select the proper 2′-deoxynucleoside triphosphate (dNTP) to form a Watson-Crick base pair despite being outnumbered by other dNTPs and similar ribonucleoside triphosphates (rNTP). In fact, replicative DNA polymerases can generate double-stranded DNA at rates of tens or hundreds of nucleotide additions per second while only incorrectly matching a nucleotide once every tens to hundreds of thousands of nucleotides added [1]. This level of specificity suggests the dynamics and conformations of DNA polymerase are important for proper substrate binding and catalysis.

**Figure 1:**
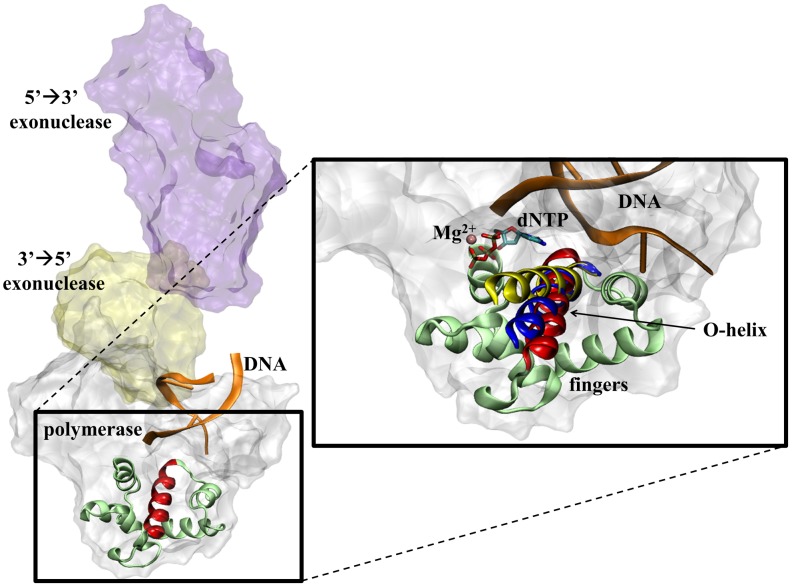
The structure of DNA polymerase I (PDB code 1TAU) bound to DNA (orange ribbons) is shown on the left depicting the 5′→3′ exonuclease (purple surface), 3′→5′ exonuclease (yellow surface), and polymerase (white surface) domains (left). The inset on the right shows a close-up of the mobile fingers subdomain (light green) of Bacillus stearothermophilus DNA polymerase I, with the open (red), ajar (blue), and closed (yellow) conformations of the O-helix shown in relation to the dNTP substrate (sticks) and Mg2+ ion (pink).

DNA polymerase I consists of 5′→3′ exonuclease, 3′→5′ exonuclease, and polymerase domains (Figure 1). The Klenow fragment of DNA polymerase I is an N-terminal deletion of the dispensible 5′→3′ exonuclease domain [2]. Within the Klenow fragment, the polymerase domain resembles the shape of a human hand with a thumb subdomain that grasps the DNA, a palm subdomain that contains the active site, and a mobile fingers subdomain involved in dNTP binding [3], [4]. The thumb (residues 496–595), palm (residue 617–655 and 830–869), and fingers (residues 656–818) subdomains of DNA polymerase were named based on their positioning around the bound DNA as observed in crystal structures. The fingers domain consists of multiple *α*-helices highlighted by the O-helix that directly interacts with the dNTP substrate upon binding. X-ray crystallography and solution kinetics studies have observed the fingers subdomain in three distinct conformations (Figure 1), which are dependent on the presence or absence of a dNTP in the active site [5], [6], [7], [8]. The fingers subdomain primarily resides in an “open” conformation with no dNTP bound (binary state) to the polymerase. Upon binding of a dNTP (ternary state) that forms a proper Watson-Crick base pair with the template strand, the fingers domain enters a “closed” conformation that helps position the substrate in the active site during catalysis [5], [9]. And recently a third “ajar” conformation was discovered that places the fingers domain in a semi-open state when a dNTP binds that forms a mismatch with the template strand [10].

The mechanism of binding reactants and subsequently releasing products post-elongation has been studied extensively, but mechanistic details for the opening and closing of the fingers domain have never been elucidated. The O-helix undergoes a ∼40° rotation when the fingers close around the bound dNTP, while the side chains of several amino acids on the helix are involved in key protein-ligand interactions. From close examination of the closed crystal structure, an arginine and lysine in the O-helix form salt bridges to help neutralize the negative charge of the triphosphate on the dNTP, while a tyrosine near the active site plays a key role in substrate specificity and closing of the fingers domain [11]. Single-molecule Förster resonance energy transfer (FRET) experiments of the DNA polymerase-DNA (binary) complex indicate that the enzyme fluctuates between the three conformations (open, ajar, and closed), but incorporation of an incorrect nucleoside causes the O-helix to undergo a ∼15° rotation relative to the open structure, causing the ajar conformation to dominate [12]. More recent DNA polymerase FRET studies suggest the open conformation is present 81% of the time with DNA bound in the absence of dNTP [13]. Although the structures of the ternary (closed and ajar) and binary (open) conformations have been characterized using X-ray crystallography, the dynamics and atomistic details of the conversions between the various states occur too quickly to observe with standard experimental techniques. In this, we report the first structural images for the conversion between closed, ajar and open binary conformations.

Computational methods can help describe the dynamics of biomolecules on an atomistic level not easily reached by experimental structural biology [14]. In particular, molecular dynamics (MD) simulations are able to simulate the movements of these molecules using Newton's classical laws of motion. Simulations of apo molecules have led to the discovery and confirmation of important biological conformational states and conformational interconversions not available to traditional experimental techniques [15], [16]. MD has been used in many studies to understand the dynamics of protein-DNA complexes, even DNA polymerase [17], [18], [19]. Specifically, Golosov et al. used targeted (i.e. biased) MD to observe the translocation of DNA after dNTP insertion by artificially steering the simulation towards the desired endpoint [20]. Unbiased MD simulations have previously been performed, but limited to understanding localized motions of the amino acids and nucleotides in the DNA polymerase complex, and have been unable to observe any large-scale biomolecular motions during short time-scales [17], [21], [22], [23], [24] except for smaller DNA polymerase complexes [25], [26]. In its infancy, MD could only be utilized for these short (picosecond to nanosecond) time-scales, but recent advances in computational hardware and MD software have made it possible to reach significantly longer time-scales into the microsecond and even millisecond range [27]. Consequently, it is now possible to computationally model domain movements that require long time-scale dynamics to observe [14].

The opening of the fingers domain in DNA polymerase I has never been studied using unbiased all-atom computational methods on the µs time-scale because of the high computational cost of modeling such a large conformational change in a large biomolecule. Biased MD simulations apply additional external forces that might unnaturally influence the dynamics, but are useful for studying conformational inter-conversions when sufficiently long unbiased trajectories cannot be simulated. When adequate computational resources are available, unbiased methods are preferred for observing conversions between structures and thus for examining biomolecular mechanistic details without biasing the dynamics. In this study, we have utilized recent computational advances to simulate the opening of the fingers domain starting from the closed (PDB 1LV5 [28]) and ajar (PDB 3HP6 [10]) conformations of *Bacillus stearothermophilus* DNA polymerase Klenow fragment using dynamics on the microsecond time scale, and also simulated the open state (PDB 1L3S [28]) for comparison. A detailed understanding of the opening process of DNA polymerase is vital as we attempt to understand the complete dynamics involved in DNA replication, and how we can apply our knowledge in biotechnology to design better polymerases for PCR. Additionally, these simulations are relevant to the polymerase community because they provide a foundation for future experimental and computational work and analysis with the ternary DNA polymerase complex. We have fully characterized the opening process that occurs prior to catalysis, and determined the key events and movements that are critical to O-helix opening. The transition from ajar to open is quick (<20 ns), while the full transition from closed to open was observed taking nearly 300 ns. We observed a key intermediate step in the pathway from closed to open involving a salt bridge between an arginine side chain on the O-helix and an aspartate in the thumb domain. We have also identified critical changes in a handful of polymerase backbone dihedrals and determined the order of events involved in the transition from closed-to-open of the fingers domain. Altogether, these simulations aid in the elucidation of the O-helix opening mechanism for DNA polymerase on an atomistic level not currently available with experimental measures.

## Methods

### Protein Preparation

Structures of *B. stearothermophilus* DNA Polymerase I were acquired from the Protein Databank with the O-helix in the open (PDB 1L3S [28]), ajar (PDB 3HP6 [10]), and closed (PDB 1LV5 [28]) conformations. Prior to simulations, the substrate ligand was removed from the active site for both the 1LV5 and 3HP6 structures to create the binary complex (enzyme+DNA) for all simulations. The 1L3S structure was crystallized with the wild-type sequence, but the 3HP6 and 1LV5 structures were crystallized with mutations. Specifically, 3HP6 was a D598A/F710Y double-mutant, while 1LV5 had a D329A mutant that required *in silico* mutations to regenerate the wild-type sequence in those structures.

### MD with Amber

The *ff99SB* force field [29] was applied to the protein along with the *parmbsc0* modifications [30] for nucleic acids. Explicit hydrogen atoms were added to all initial X-ray crystal structures using the *tleap* module of AmberTools [31]. *tleap* was also used to neutralize each system with Na^+^ counter ions and solvate a truncated octahedron unit cell with TIP3P water molecules [32] using a 12.0 Å solvent buffer between the solute and the closest edge of the unit cell for a total atom count of ∼80,000. The GPU-accelerated *pmemd* code [33] of Amber 12 [31] was used to perform all steps of MD for each system. All initial structures underwent a seven-step minimization procedure involving 1000 steps of steepest descent minimization followed by 4000 steps of conjugate gradient minimization at each step. Positional restraints on all solute heavy atoms were initially set to 10.0 kcal/mol/Å^2^ and systematically lowered during each stage down to zero for the final stage. After minimization, each system was heated linearly from 10 K to 335 K over 2.0 ns, while positional restraints were held constant at 10.0 kcal/mol/Å^2^ on the protein and DNA strands. The final stage of the preliminary equilibration process involved running MD at constant temperature (335 K) for 3.5 ns, beginning with 10.0 kcal/mol/Å^2^ positional restraints on all heavy atoms of the protein and DNA for the first 0.5 ns, and systematically lowering the restraints every 0.5 ns until reaching a final restraint weight of zero (unrestrained) over the final 0.5 ns. After this equilibration protocol, unrestrained MD was performed on all solvated systems at constant pressure (1 atm) using a Berendsen thermostat with isotropic position scaling and constant temperature (335 K) maintained with a Langevin thermostat [34] using periodic boundary conditions, saving the coordinates, velocities, and energies every 100 ps. Long-range interactions were treated with the Particle Mesh Ewald method for periodic boundaries using a nonbonded cutoff of 9.0 Å and the nonbonded list was updated every 25 steps (default). New random number seeds were chosen every 25 ns for each simulation to prevent simulation synchronization of the trajectories [35]. The SHAKE algorithm [36] was used to fix all covalent bond distances involving hydrogen, allowing a 2-fs time-step for dynamics. Given the time-scale of the expected conformational change, a mass repartitioning method [37] was used on the 1LV5 system to create hydrogen atoms three times heavier than normal hydrogen atom mass, which was compensated by lowering the mass of each heavy atom attached to any hydrogen atom in the system to maintain the same overall mass. The mass repartitioning method allowed us to increase the MD step size from 2 fs to 4 fs for the 1LV5 system. The 1L3S, 3HP6, and 1LV5 starting structures were simulated without restraints for a total of 500 ns, 1.0 µs, and 3.0 µs, respectively, which were used for all analyses.

### MD with Desmond

The 1L3S, 3HP6, and 1LV5 PDBs were prepared similarly for MD using the Desmond 3.1 MD package [38], [39], [40]. Each complex was checked for structural correctness using the Protein Preparation Wizard in Schrödinger's Maestro v9.4. Sodium and chloride ions were added to reach a final concentration of 150 mM Na^+^ (while still maintaining a neutrally charged unit cell) and the system was solvated with TIP3P water molecules after reorientation to minimize the volume in an orthorhombic box. The Amber force fields were applied to these periodic systems. Additionally, each PDB system was simulated using the Charmm27 force field [11], [41], although the Charmm36 force field has been available since 2012. The default Desmond minimization and equilibration procedure was followed, except the maximum number of steps for steepest descent and total minimization were increased to 1000 and 5000 steps, respectively. Simulations were kept at constant pressure (1 atom) and temperature (335 K) maintained with a Berendsen barostat and thermostat, respectively [42]. SHAKE was applied to all systems allowing a 2-fs time-step. Long-range interactions were treated with the Particle Mesh Ewald method for periodic boundaries using a nonbonded cutoff of 9.0 Å and the nonbonded list was updated frequently using the default settings. Coordinates and energies for the Amber ff99SB force field simulations were saved every 100 ps for a total of 500 ns, 1.0 µs, and 1.0 µs for the 1L3S, 3HP6, and 1LV5 systems, respectively. Coordinates and energies for the Charmm27 force field were saved at the same interval for a total of 500 ns for the 1L3S simulation, while the 3HP6 and 1LV5 systems were each simulated for 1.0 µs.

### MD Analysis

All MD analysis (e.g. distance/angle measurements, RMSDs, etc.) was performed using the *cpptraj* module [43] of AmberTools 13. All Desmond trajectories were centered, imaged, and converted to DCD binary trajectory file format using VMD v.1.9.1 [44] to ensure their readability by *cpptraj*.

## Results

### Opening Mechanism

In this study we performed nine MD simulations of *B. stearothermophilus* DNA polymerase I in the binary state (enzyme+DNA) starting from the open (PDB 1L3S), ajar (PDB 3HP6), and closed (PDB 1LV5) conformations of the fingers domain for a combined total of 9.5 µs. We observed DNA polymerase transition fully from the closed to open conformations starting from the 1LV5 PDB (closed) structure (see movie in the Supporting Information). We constructed the binary conformation by removing the dNTP *in silico* from the active site and performed MD using two different software packages and two unique force fields to describe the dynamics (Table 1). This transition from closed to open has never previously been observed experimentally or computationally without applying a biasing potential. As expected, the simulations were not identical (in particular, with regards to the timing of the opening process); however, they all appear to have traversed similar pathways.

**Table 1:** Summary of simulations performed on *B. stearothermophilus* DNA polymerase I in the binary complex.

No.	PDB code	Starts	Software/Force field	Results (fingers domain)	Simulation Time (µs)
1	1L3S	Open	Desmond/Charmm27	Remains open	0.5
2	3HP6	Ajar	Desmond/Charmm27	Opens after <5 ns	1.0
3	1LV5	Closed	Desmond/Charm27	Opens after ∼290 ns	1.0
4	1L3S	Open	Desmond/ff99SB	Remains open	0.5
5	3HP6	Ajar	Desmond/ff99SB	Opens after <5 ns	1.0
6	1LV5	Closed	Desmond/ff99SB	Opens after ∼800 ns	1.0
7	1L3S	Open	Amber/ff99SB	Remains open	0.5
8	3HP6	Ajar	Amber/ff99SB	Opens after <50 ns	1.0
9	1LV5	Closed	Amber/ff99Sb	Opens after ∼1.9 µs	3.0

**Figure 2:**
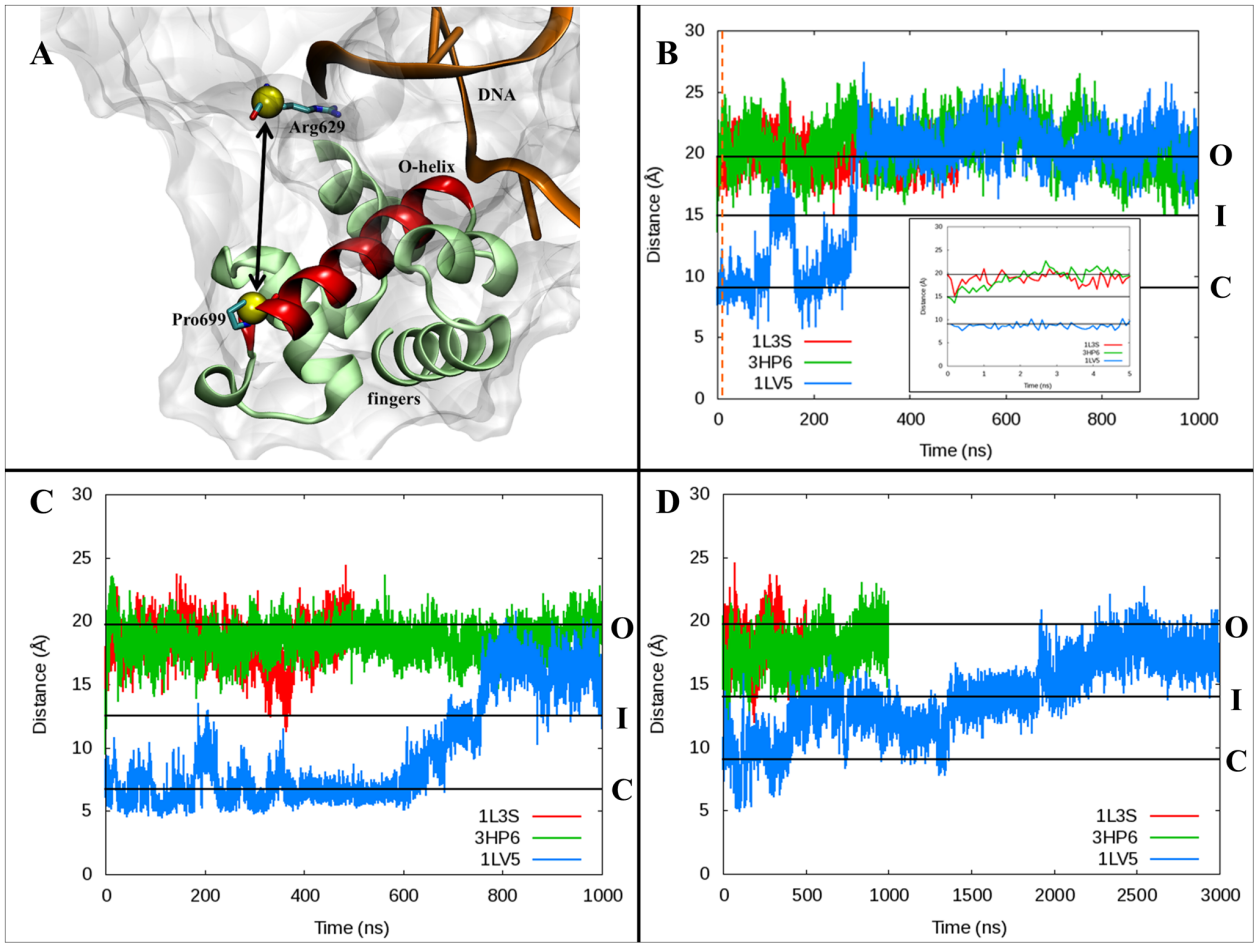
The dynamics of the fingers domain illustrated by A) the α-C distance between Pro699-Arg629 of DNA polymerase simulated with B) Desmond using the Charmm27 force field, C) Desmond using the Amber ff99Sb force field, and D) Amber using the Amber ff99SB force field. The simulations are named according to their PDB codes and initial starting conformations where 1L3S (red) began in the open conformation, 3HP6 (green) began in the ajar conformation, and 1LV5 (blue) was started from the closed conformation. The black horizontal lines represent the distances corresponding to the three major observed conformations: open (O), closed (C), and the newly observed intermediate (I) state. The inset in B) displays a close-up of the distances from 0–5 ns (highlighted by the vertical orange dashed line) to more clearly depict the relatively quick opening of the simulation started from the 3HP6 ajar conformation.

**Figure 3:**
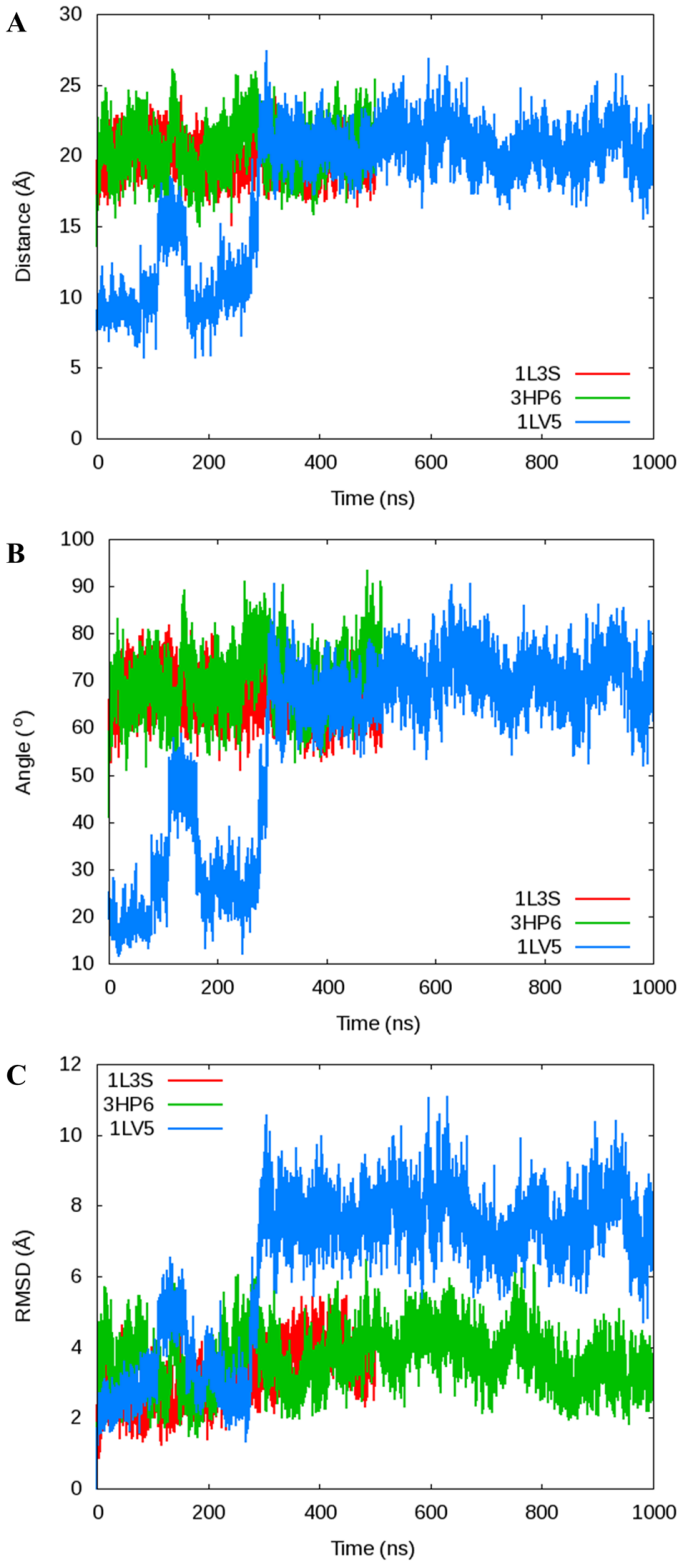
Comparison of two different methods for measuring the opening/closing of the O-helix on DNA Polymerase I. A) The *α*-C distance between Arg629 and Pro699 shown in this manuscript compared to B) the angle between the *α*-C of Arg629, Gly711, and Asn700 used by Golosov et al. to determine the conformation of the O-helix and C) a plot of the RMSD of the fingers domain as a function of time in reference to the original crystal structure used to start each simulation. The distance, angle, and RMSD measurements are directly comparable, validating our use of the Arg629-Pro699 distance.

**Figure 4:**
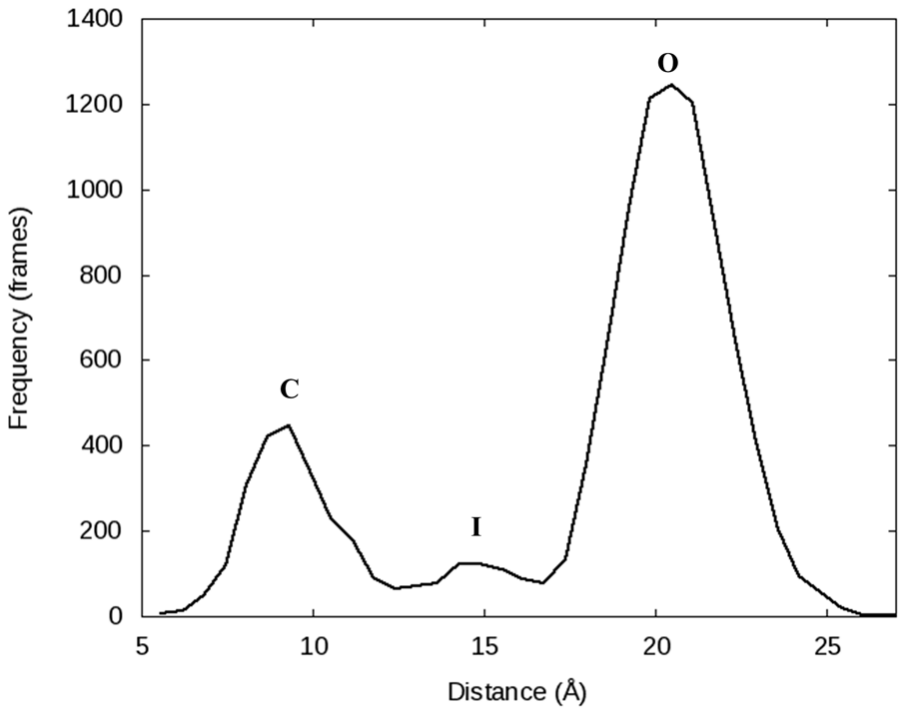
A histogram of the Pro699-Arg629 *α*-C distances for the 1.0 µs simulation (10,000 frames total) of PDB 1LV5 performed with Desmond using the Charmm27 force field. The histogram clearly shows three distinct conformations were sampled: closed (C), open (O), and the newly observed intermediate (I) state.

To describe the conformation of the fingers domain at any given time, we measured the distance between the *α*-carbons of Pro699 at the end of the O-helix and Arg629 residue in the thumb domain of DNA polymerase (See Figure 2A). This single distance is able to successfully capture the movements of the fingers domain as well as an angle used in a publication by Golosov et al. [20] and a plot of the RMSD of the fingers domain as a function of time in reference to the original crystal structure used to start each simulation (Figure 3). The plot of the Pro699-Arg629 distance (Figure 2B) illustrates the dynamics of the fingers domain for each simulation using the Desmond MD package with the Charmm27 force field (See Figure 2C and 2D for corresponding plots with the Amber ff99SB force field). The 1L3S (open) simulation remains in the open conformation for the entire 500 ns trajectory. 3HP6 (ajar) begins in the ajar conformation, but very quickly (<5 ns) opens to a distance corresponding to the open conformation. Meanwhile, the fingers domain for the 1LV5 (closed) simulation is initially closed for more than 100 ns, but partially opens into a conformation similar to but distinct from the ajar shortly at ∼125 ns. The polymerase remains in this intermediate state for ∼50 ns before it returns the closed state for ∼100 ns duration and finally fully opens at ∼290 ns where it persists for the remainder of the simulation. The ability of the DNA polymerase fingers domain to clearly sample all three conformations (Figure 4 and Table 2) coincides with experimental evidence that suggests each state is thermodynamically accessible in the binary state [13].

**Table 2:** An analysis of the percent of time that each DNA Polymerase simulation spent in the closed, intermediate, and open conformations.

No.	Initial Conformation	Percent Closed (%)	Percent Intermediate (%)	Percent Open (%)	Simulation Time (µs)
1	Open	0%	0%	100%	0.5
2	Ajar	0%	0.2%	99.8%	1.0
3	Closed	22.5%	6.5%	71.0%	1.0
4	Open	0%	0%	100%	0.5
5	Ajar	0%	0.4%	99.6%	1.0
6	Closed	75.7%	3.4%	20.9%	1.0
7	Open	0%	0%	100%	0.5
8	Ajar	0%	4.4%	95.6%	1.0
9	Closed	34.8%	28.9%	36.3%	3.0

Refer to Table 1 for more information about each simulation.

We have utilized two of the most recognized and accurate MD force fields (Charmm27 and Amber ff99SB) available for studying the motions of biological macromolecules [45]. By applying multiple force fields and MD software packages (Desmond and Amber) with unique sampling algorithms we tested the dependence of observed structural changes on the methodology used. We focus here mostly on the results of the dynamics simulated with Desmond using the Charmm27 force field because the observed conformational changes occurred over a shorter time-scale making the analysis simple and well-defined. The dynamics utilizing Amber ff99SB simulated with both Desmond and Amber MD software showed similar overall patterns to the Charmm27 force field, and are also represented in the Figure 2.

The results of the six simulations using the Amber ff99SB force field (Figure 2C and 2D) are summarized here as they relate to the Charmm27 force field simulations (Figure 2A). Using the Desmond software and the Amber ff99SB force field, 1L3S remained in the open conformation for the entire simulation whereas the 3HP6 (ajar) simulation transitioned very quickly (<5 ns) to the open conformation and remained there. The 1LV5 (closed) Amber ff99SB simulation also transitioned into the open conformation, but this process took longer (∼775 ns) than it did with the Charmm27 force field (∼290 ns). Using the same starting structure and simulating with Desmond and the Amber ff99SB force field, the polymerase was primarily in the closed conformation until ∼600 ns, and underwent a relatively slow and steady transition (∼175 ns) into the open conformation and never re-visited the closed conformation after leaving it (Figure 2C). With the Amber MD software using the ff99SB force field, 1LV5 did not move to the open conformation until nearly 2.0 µs of simulation time (Figure 2D). The apparent time-dependence of these simulations is likely created by the inherent differences between the two force fields; the Amber ff99SB force field does not allow the polymerase to be as dynamic as the Charmm27 force field. Additionally, there is likely some fluctuation in the timing of the opening transition created by the nature of MD simulations that must traverse complicated potential energy surfaces (PES) utilizing different initial seeds/velocities, causing the simulated timing of conformational changes to vary from one trajectory to another. Thus, the timing of these transitions is likely not well defined and should not be considered the true amount of time required for each conformational change.

**Figure 5:**
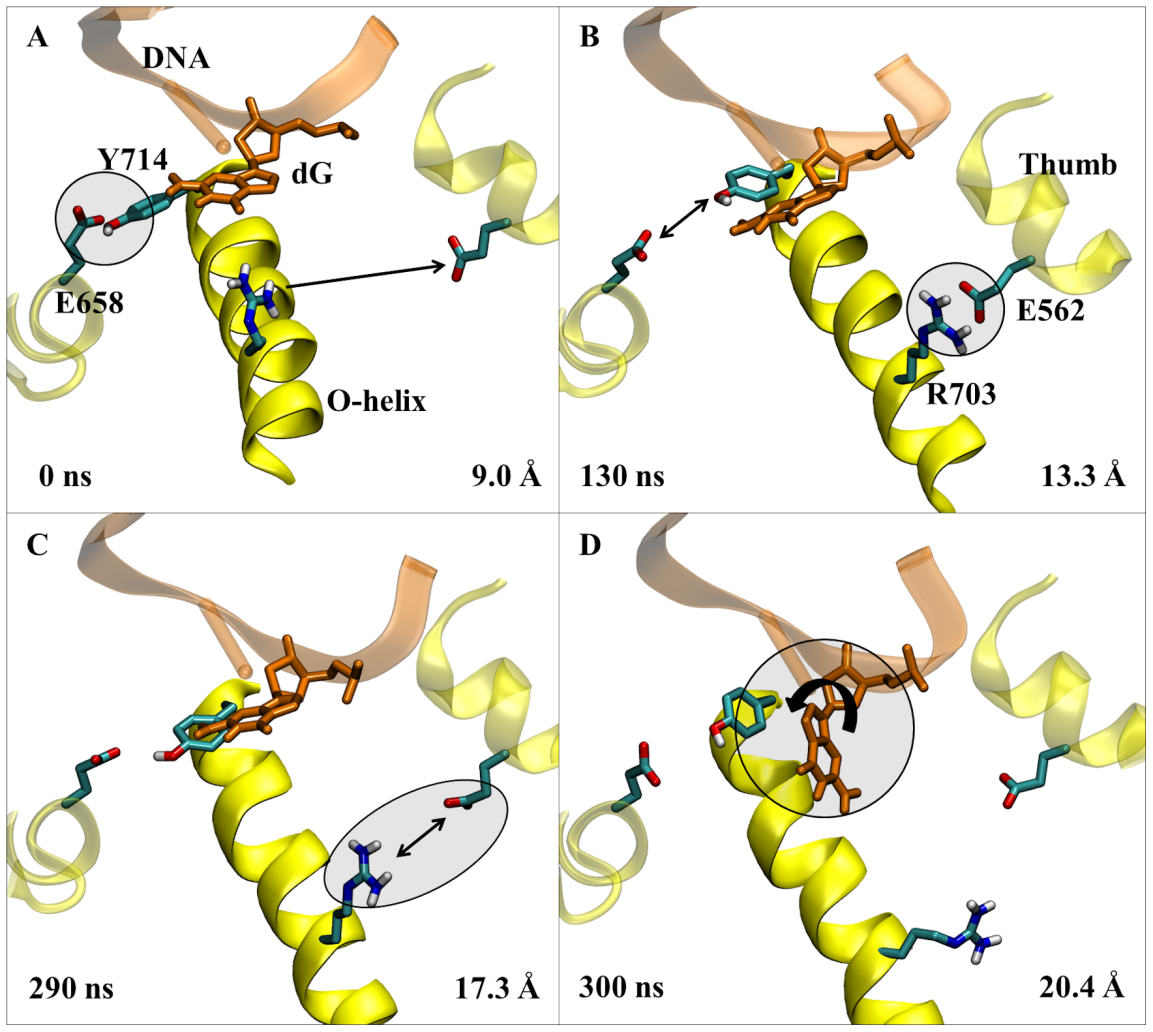
The proposed opening mechanism for the fingers domain for DNA polymerase I. The secondary structure of the relevant polymerase residues including the O-helix are shown in yellow ribbons, while the DNA is shown in orange. The key event in each image is circled. A) The X-ray crystal structure of PDB 1LV5. B). The intermediate state showing the breaking of the Tyr714-Glu658 hydrogen bond, and the formation of the salt bridge between Arg703 and Glu562. C) Depiction of the breaking of the Arg703-Glu562 salt bridge, which is quickly followed by D) the rotation of the N-β-glycosyl bond of the template nucleotide allowing Tyr714 to replace the base in the active site, and resulting in the fully open conformation of DNA polymerase I. Simulation times and O-helix distances correspond to the 1LV5 simulation performed using Desmond and the Charmm27 force field.

The detailed motions of DNA polymerase during the transition from closed to open observed with Charmm27 are shown in Figure 5. The 1LV5 (closed) crystal structure showed the existence of a hydrogen bond between the side chains of Tyr714 and Glu658 in the ternary (enzyme-DNA-dNTP) state (Figure 5A), but after removing the dNTP and simulating the closed structure this hydrogen bond is quickly broken (Figure 5B). This allows Tyr714 to move toward the template DNA base and causes the O-helix to open slightly (∼1.5 Å). The O-helix is held in this intermediate position by a salt bridge between Arg703 and Glu562 of the thumb subdomain, while Tyr714 and the guanine in the DNA template continue to compete for the insertion site (Figure 5B). Shortly after the Arg703-Glu562 salt bridge interaction is broken (Figure 5C) the O-helix opens further, pulling Tyr714 into the insertion site, inducing a rotation of the *N*-*β*-glycosyl bond of the template nucleotide, and moving the template base out of the active site (Figure 5D). The precise ordering of the last two steps is not fully established because different force fields yielded different results. The Charmm27 force field predicted the salt bridge to break prior to the *N*-*β*-glycosyl bond rotation, while the two Amber ff99SB force field simulations suggested the opposite ordering. However, in all three simulations the steps succeeding the intermediate conformation (Figure 5B) appear closely correlated implying that they may occur nearly simultaneously.

### Key Intermediate Conformation

Examination of the fingers domain dynamics in the 1LV5 (closed) simulation revealed an intermediate state corresponding to a Pro699-Arg629 distance of ∼13–15 Å that is stable along the pathway from closed to open from ∼100–170 ns and again from ∼280–290 ns (Figure 2B). This state is not identical to the ajar state of the 3HP6 crystal structure although the observed Pro699-Arg629 distances are similar (see below for more details).

The intermediate conformation (Figure 5B) provides key insight into the opening process of DNA polymerase I. This pathway has never been observed crystallographically, likely due to the complexity of trapping such a short-lived intermediate in the binary state. However, the existence of this conformation utilizing two different MD packages (Desmond and Amber) and two different force fields (Charmm27 and Amber ff99SB) known to describe well the protein and DNA systems indicates that its presence in the opening mechanism is not dependent on the computational method and strongly supports the existence of this intermediate during opening of the fingers domain. For the 1LV5 (closed) Desmond simulation using the Charmm27 force field, the intermediate persisted for a total of ∼80 ns before the fingers domain opened fully. The intermediate was longer-lived using the Amber ff99SB force field (∼400 ns and ∼1.0 µs lifetimes using the Desmond and Amber MD software, respectively), although this could be expected since the dynamics appear to move quicker using the Charmm27 force field in general for DNA polymerase I, as previously mentioned in the Results section.

**Figure 6:**
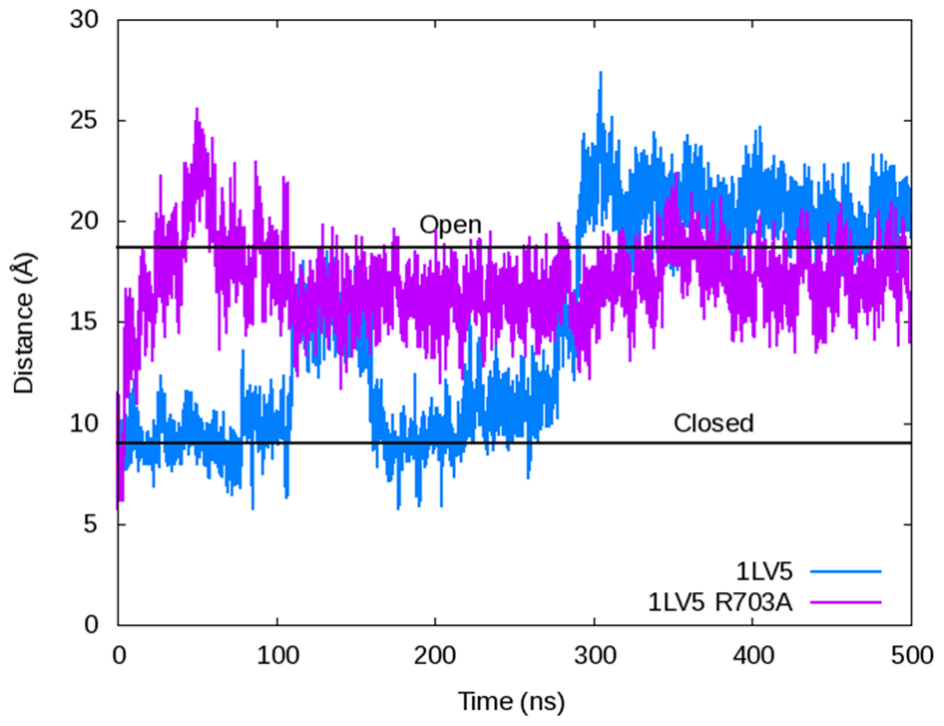
The O-helix distance as measured by the α-C distance between Arg629 and Pro699 depicting the opening of the fingers domain for the wild-type 1LV5 (blue) and R703A mutant (purple) simulated using Desmond and the Charmm27 force field. The plot shows the mutant reaching the open conformation in <50 ns, while the wild-type does not open fully until ∼290 ns.

The intermediate state observed in the 1LV5 (closed) simulation is stabilized by a key salt bridge between an arginine residue in the O-helix and a glutamate residue in the thumb domain of DNA polymerase. To test whether the salt bridge constitutes a substantial obstacle for opening, we *in silico* mutated Arg703 to an alanine residue in 1LV5 and re-started the simulation under the same conditions and simulated for 500 ns. The fingers domain of the R703A mutant opened in <50 ns, while the wild-type required ∼290 ns to reach the same conformation (Figure 6). Given that the only difference between these two starting structures is the mutation from arginine to alanine at position 703, this result provides further evidence of the importance of the Arg703-Glu562 salt bridge intermediate along the opening pathway for the fingers domain of DNA polymerase. The arginine residue is highly conserved in bacterial DNA polymerase I enzymes. 28 out of 33 DNA polymerase I enzyme sequences from bacteria in UniProt contained an arginine at this location in the O-helix, including ones from *Escherichia coli* and *Thermus aquaticus* (*Taq*), which have been structurally characterized. Arg703 is also known to be important for polymerase activity in bacteria [46]. Mutation studies of the corresponding arginine in *Taq* DNA polymerase I showed a clear loss of polymerase function when mutated, although the role of the arginine residue was not described [46]. Our simulations support those mutagenesis studies, indicating the importance of this arginine to the polymerase and additionally illustrate its role in forming a key intermediate during the opening of the fingers domain.

**Figure 7:**
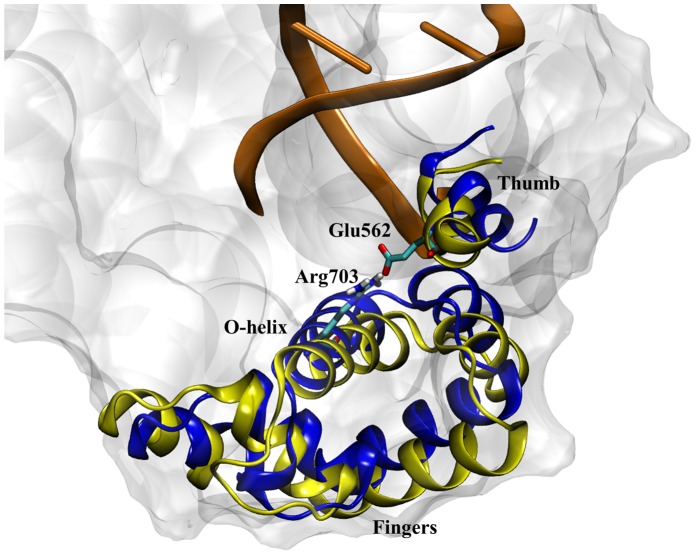
An overlay of the DNA polymerase fingers subdomain for the 3HP6 crystal structure (blue ribbons) and the observed 1LV5 intermediate state (yellow ribbons) characterized by the Arg703-Glu562 salt bridge. Although the end of the O-helix for both structures is near the same location, the α-helices in the fingers domain are clearly different resulting in the 4.3 Å RMSD between the 1LV5 intermediate and the 3HP6 crystal structure.

**Figure 8:**
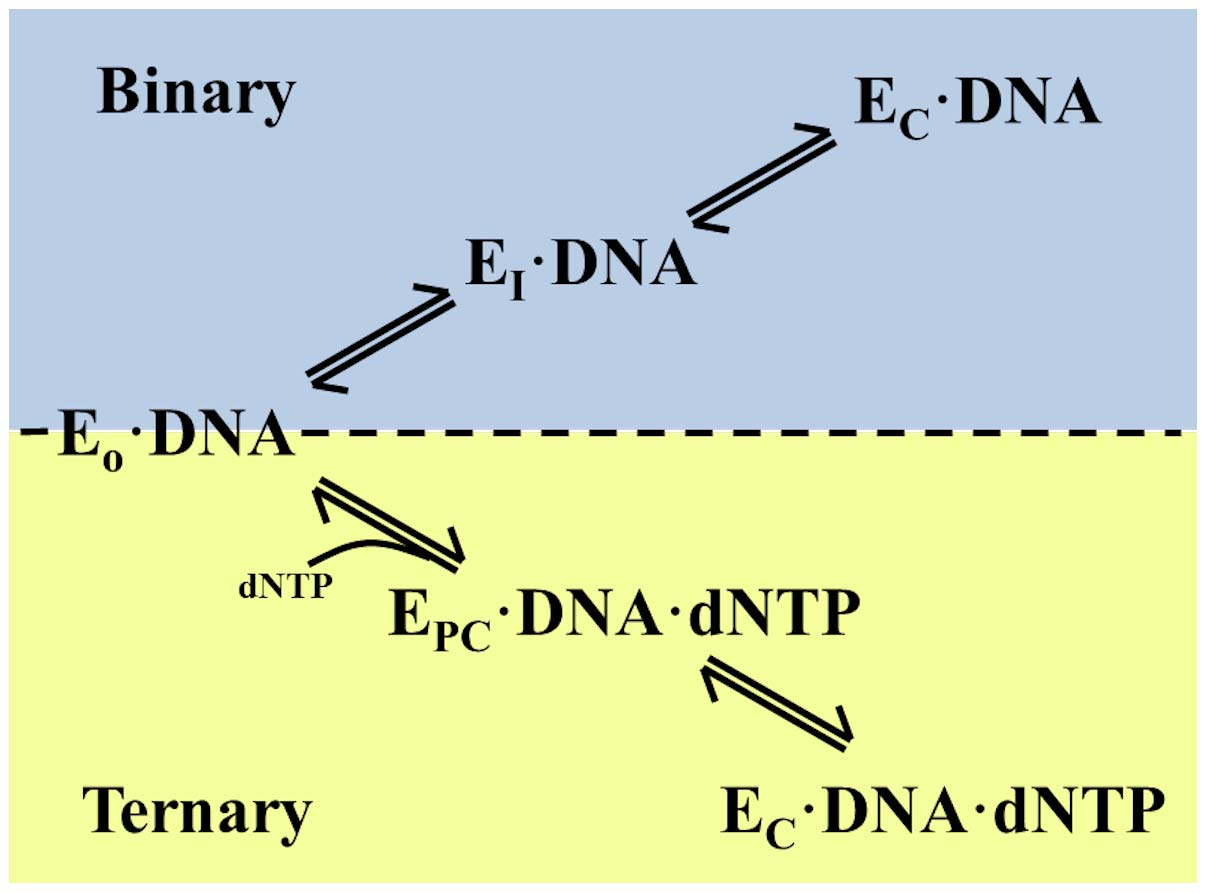
The proposed pathway for opening and closing of DNA polymerase I in the presence and absence of dNTP. In the binary complex (blue), the polymerase transitions through the intermediate observed in this study (EI•DNA), while the ternary complex (yellow) transition is a separate, partially-closed conformation (EPC•DNA•dNTP) on its way to the closed conformation. This pathway depicts the enzyme in two different “ajar” conformation (EI or EPC) determined by the presence or absence of dNTP in the active site.

As a final note on the intermediate state, although the fingers domain is clearly between the closed and open conformations, this newly observed state is not identical to the ajar state observed in the 3HP6 PDB structure. The simulated intermediate has a heavy atom root-mean-square deviation (RMSD) of 4.3 Å from the 3HP6 crystal structure. The largest structural differences between the intermediate and the 3HP6 crystal structure arise in the fingers subdomain with Arg703 and the thumb region of the polymerase where Glu562 resides (Figure 7). The Arg703-Glu562 salt bridge is not present in the 3HP6 crystal structure nor does it ever exist in any of the simulations starting from the 3HP6 ajar conformation. The 3HP6 crystal structure was generated by trapping DNA polymerase with a non-Watson Crick dNTP paired to the template strand in the active site, while our simulations are performed in the absence of a dNTP molecule to mimic the dynamics of the protein after elongation of the DNA primer strand has occurred. This means that, experimentally, the two “ajar” conformations reside on two different potential energy surfaces where the 3HP6 ajar state is only observed in the presence of a dNTP in the active site, while the proposed intermediate state is present only in the absence of dNTP (Figure 8). This is contrary to the literature reported prior to this study that assumed the polymerase conformation observed in the 3HP6 crystal structure was identical to the conformation of polymerase observed in the absence of dNTP. The single-molecule FRET experiments [13] that previously reported the presence of open, ajar, and closed conformations in the binary state probably observed the intermediate proposed in this study instead of the ajar state documented from X-ray crystallography that likely only occurs with a bound mismatch dNTP. Thus, the hypothesis for this new intermediate structure from MD is consistent with solution studies that show an intermediate state between the open and closed conformations in the absence of dNTP. The exact purpose of the intermediate is not fully understood yet, but it is clear that the presence of the intermediate slows the transition from the closed to the open conformation in the binary complex. Based on the similarity between binary and ternary pathways to the closed conformation (Figure 8), we speculate that the intermediate may also play a role in the closing of the fingers domain during dNTP binding, possibly providing an energetic barrier to opening that aids the enzyme during substrate recognition.

### Important Dihedrals Involved in Opening

**Figure 9:**
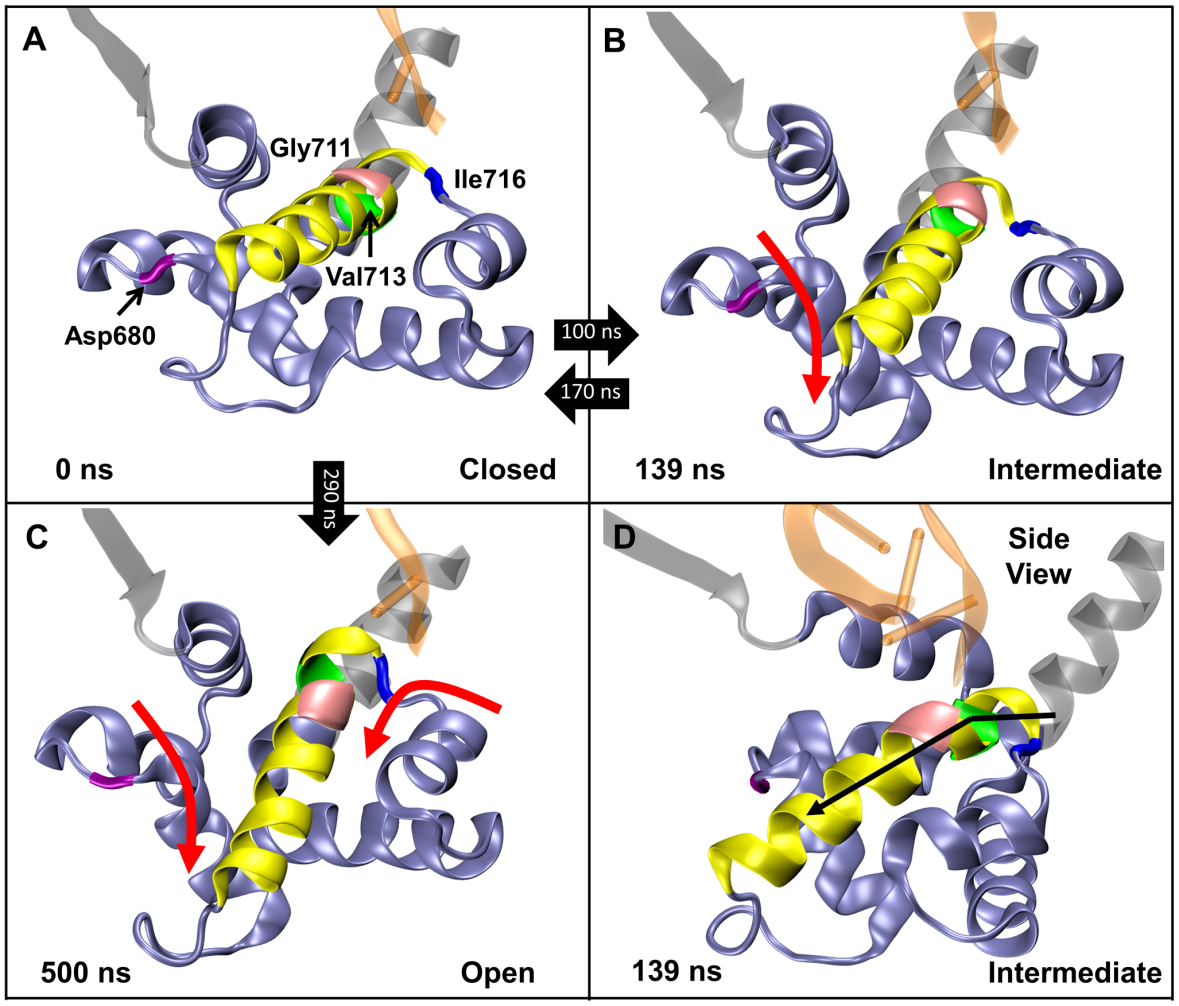
A depiction of the residues with backbone dihedrals—Asp680φ (purple), Gly711φ (pink), Val713ψ (green), and Ile716φ (blue)—identified as important in the finger domain opening process of DNA polymerase. The fingers domain is shown in an ice blue cartoon representation, while the O-helix is shown in yellow cartoon. The times within the black arrows between panels indicate the transition times between the conformations. A) Conformation of the fingers domain in the 1LV5 crystal structure (closed) prior to running MD. B) Representative conformation of the intermediate state observed from ∼100–170 ns (139 ns shown) of simulation time. The red arrow indicates the large-scale motion of the N-helix due to a rotation about the Asp680φ dihedral. C) Representative conformation of the open state observed from ∼290–1000 ns (500 ns shown) of MD caused by a rotation of the Asp680φ and Ile716φ dihedrals. D) A side view of the intermediate state at 139 ns depicting the bend in the O-helix caused by rotations of the Gly711φ and Val713ψ dihedrals.

**Figure 10:**
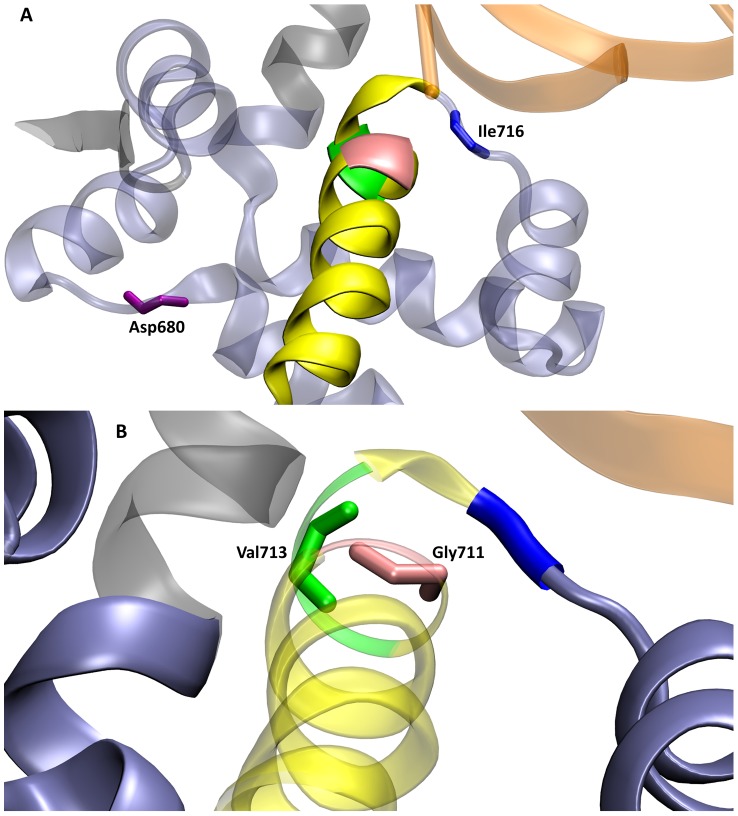
A close view of the exact orientations of the important dihedrals from the closed (1LV5) crystal structure for A) Asp680φ (purple) and Ile716φ (blue) and B) Gly711φ (pink) and Val713ψ (green). The fingers domain is shown in an ice blue cartoon representation, while the O-helix is shown in yellow cartoon.

Each backbone dihedral in the fingers subdomain of the simulation started from the closed conformation (1LV5) was compared to the corresponding open (1L3S), ajar (3HP6), and closed (1LV5) crystal structure values. This investigation revealed four specific backbone torsions important for opening of the fingers domain—Asp680φ, Gly711φ, Val713ψ, and Ile716φ (Figure 9). These dihedrals were identified because each dihedral rotation corresponds to a significant change in the structure of the fingers domain involved in converting between the open, ajar, and closed conformations. The original rotation of each dihedral in the closed (1LV5) crystal structure is shown in Figure 10. In the open crystal structure (1L3S) the Asp680φ, Gly711φ, Val713ψ, and Ile716φ dihedrals have values of −74.5°, −61.3°, −33.8°, and −141.7°, respectively. According to the ajar crystal structure, the Gly711φ and Val713ψ dihedrals rotate by ∼11° and ∼7°, respectively, during an ajar-to-open transition. Meanwhile, the Asp680φ and Ile716φ values undergo significant (∼25° and ∼60°, respectively) transitions themselves between the closed (1LV5) and open (1L3S) crystal structures. These changes were all observed during our simulation that began in the closed and transitioned to the fully open conformation.

**Figure 11:**
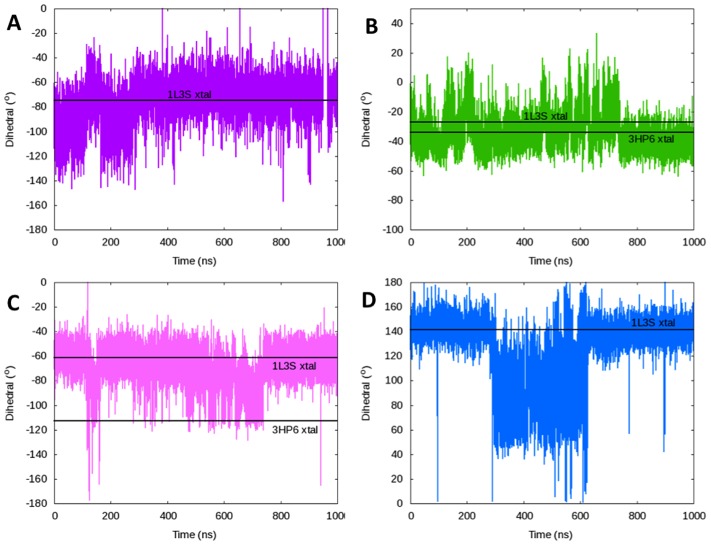
The relative dihedral values as a function of simulation time for the backbone torsions determined to be key to the fingers domain of DNA polymerase transitioning from the closed to open conformations—A) Asp680φ, B) Gly711φ, C) Val713ψ, and D) Ile716φ. Solid black lines indicate the values of each dihedral in the pertinent crystal structures, where 1L3S is in the open state and 3HP6 is the ajar conformation.

Close examination of the dihedral values as the simulation progresses (Figure 11) shows the ordering and impact of each dihedral. The transition from the closed state (Figure 9A) to the intermediate (Figure 9B) is initiated by the ∼30° rotation of the Asp680φ dihedral at ∼100 ns (Figure 11A), which results in a large-scale movement of the N-helix in the fingers domain. Subsequently, the Gly711φ and Val713ψ dihedrals rotate by ∼20° and ∼35° (Figure 11B–C), respectively, creating a bend in the O-helix (Figure 9D). In the Desmond/Charmm27 simulation the fingers domain transitions back into the closed conformation after ∼170 ns. Between 280–290 ns, the fingers domain undergoes two major dihedral rotations to complete the transition to the open conformation (Figure 9C). Once again, the process is initiated by the rotation about the Asp680φ dihedral (lowering the N-helix), followed shortly by a ∼60° rotation of the Ile716φ dihedral (Figure 11D). In this case, the rotation of the Asp680φ dihedral is enough to overcome the barrier necessary to rotate the Ile716φ dihedral and reach the fully open state.

**Figure 12:**
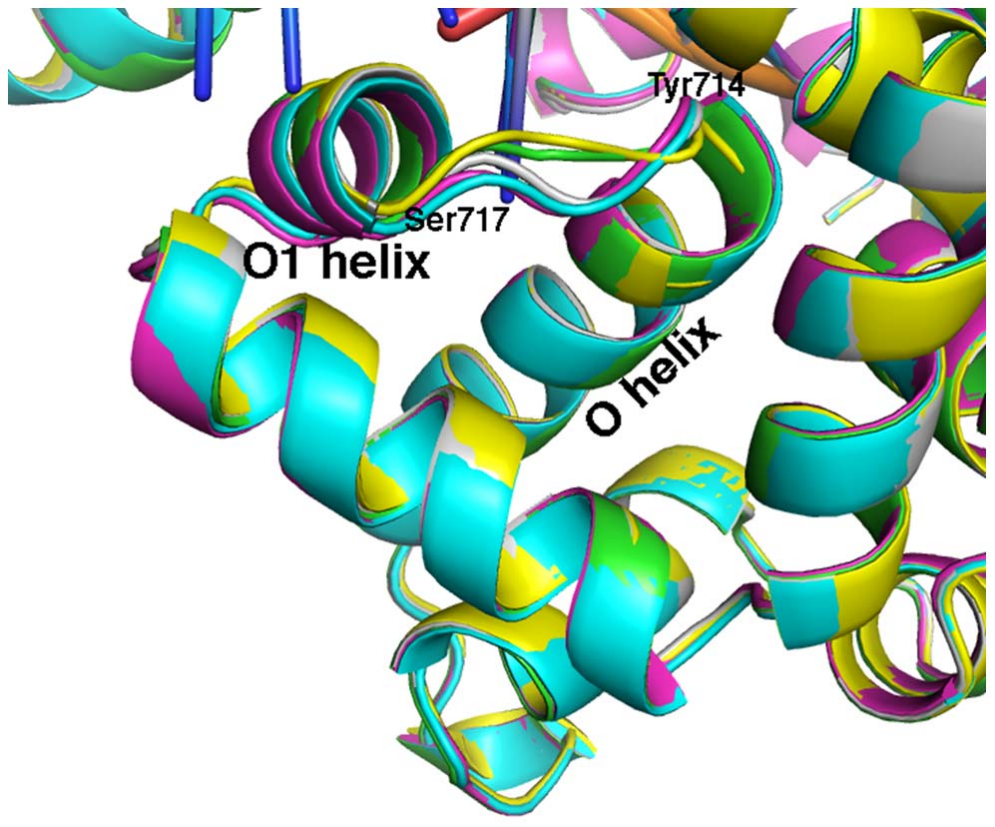
Bacillus DNA polymerase-DNA complexes before and after processive DNA synthesis in the crystal. The fingers subdomain is shown before (1L3S.pdb, cyan), and after the incorporation of 1 (1L3T.pdb, green), 2 (1L3U.pdb, magenta), 3 (1L5U.pdb, gray), and 6 (1L3V.pdb, yellow) nucleotides into the DNA (crystal structures from [7]).

The dihedrals from the simulations appear to correlate well with the values from the existing crystal structures for each state (Figure 9). Of interest, though, is the observation that although the fingers domain appears fully open after 290 ns, the Gly711φ, Val713ψ, and Ile716φ all make substantial (≥20°) transitions between 600 and 725 ns producing structures in excellent agreement with the experimental values. The ∼60° rotation about the Ile716φ dihedral actually coincides with the movement of the template DNA base flipping out of the pre-insertion site and back into the active site (where it resided in the 1LV5 closed crystal structure). The rotations by Gly711φ and Val713ψ correspond to a rotation of the Tyr710χ_1_ dihedral so the tyrosine side chain is positioned for better π-stacking with the nucleotide of the template DNA base. The dynamical nature of this region of the O-helix is consistent with structural heterogeneity in crystal structures of open, binary complexes of Bacillus DNA polymerase before and after catalyzing DNA synthesis [7]. While the overall structure of the enzyme remains the same, the structure of the loop between the O and O1 helices (residues 714–717) flips back and forth between two states after each step of processive DNA synthesis in the crystal (Figure 12), suggesting this region near Val713 and Ile716 is flexible. Although the final orientation of Tyr710 and the template DNA base is not consistent with the original 1L3S open crystal structure, this movement hints at the fundamental dynamics of the DNA polymerase active site while in the open state. Based on these simulations, we can conclude that the template base entering the active site is energetically accessible while polymerase is in the open state. Currently, it is unknown where the template base recognizes an incoming dNTP, although it has been hypothesized the preliminary interactions occur outside the active site [47]. These simulations suggest that the template DNA base could enter the active site prior to dNTP binding and recognize the incoming base while already in the active site instead of outside the active site.

### Dynamics of the Tyr714/Glu658 Motif

**Figure 13:**
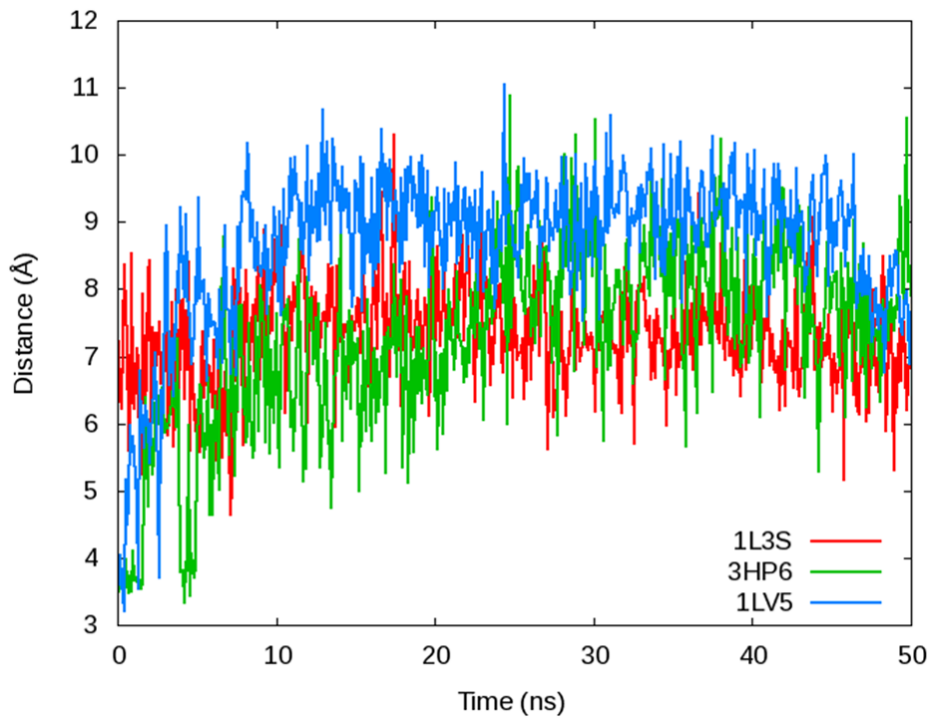
The distance between the side chain OH on Tyr714 and δ-C of Glu658 for the open (1L3S, red), ajar (3HP6, green), and closed (1LV5, blue) structured simulated using Desmond with the Charmm27 force field. Tyr714 and Glu658 are not hydrogen bonded in the 1L3S PDB (corresponding to a distance of 6.3 Å) or during any of the 1L3S simulation. The two residues are hydrogen bonded in the initial 3HP6 and 1LV5 PDB structures (distance <4 Å), but the hydrogen bond is broken within first 5.0 ns of each simulation and does not reform. Note that this figure has been scaled to only the first 50 ns of simulation time to demonstrate the timing of the Tyr714-Glu658 hydrogen bond breaking.

The Tyr714/Glu658 motif of DNA polymerase plays a vital role in dNTP binding and stability of the ternary complex due to its position in the active site. The 1LV5 and 3HP6 crystal structure suggests a stabilizing hydrogen bond between the side chains of Tyr714 and Glu658 for the ternary complex in the closed and ajar conformations respectively, while no hydrogen bond is expected for the open, binary state based on the 1L3S crystal structure. Upon removing the dNTP in the 1LV5 and 3HP6 structures, the Tyr714-Glu658 hydrogen bond is broken quickly (<5 ns, see Figure 13), suggesting this is the initial step toward opening of the fingers domain. Furthermore, recent studies have suggested this hydrogen bond plays only a minor role in stability of the ternary complex [13], so it is not surprising the hydrogen bond is not present for long.

**Figure 14:**
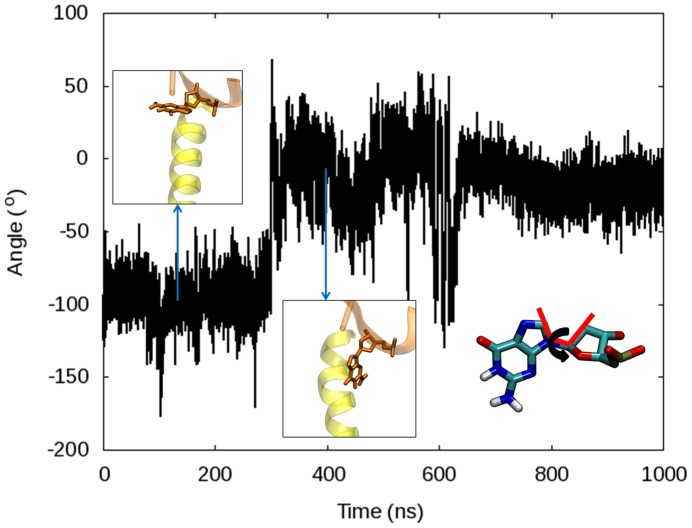
The relative degree of rotation of the *N*-*β*-glycosyl bond for the template nucleotide in the 1LV5 simulation performed with Desmond using Charmm27 force field. The torsion corresponds to an angle of roughly −90° when the nucleotide is in the active site and then changes to ∼0° when the *N*-*β*-glycosyl bond rotates moving the template nucleotide out of the active site entirely. Representative conformations of the nucleotide are shown at 130 ns and 400 ns to show the rotation. The torsion being measured is defined in the bottom right of the figure.

According to crystal structures, the position of Tyr714 in the active site changes substantially based on the state of DNA polymerase. In the ternary complex with the fingers domain closed (1LV5) or ajar (3HP6), Tyr714 is positioned next to the template base and hydrogen bonded to Glu658. In the binary state (1L3S), Tyr714 moves into the active site, taking the place of the template nucleotide. In all of the 1LV5 and 3HP6 simulations, after early disruption of the Tyr714-Glu568 hydrogen bond, Tyr714 becomes more mobile creating van der Waals contacts with the template base. Eventually, these clashes result in a ∼90° rotation of the *N*-*β*-glycosyl bond (Figure 14) of the nucleotide moving the nucleotide out of the active site entirely, while Tyr714 replaces the nucleotide in the active site and begins π-stacking with the *n-1* base on the template strand, as the 1L3S (open) PDB structure suggested. For the 1LV5 Desmond simulation performed with the Charmm27 force field, this transition occurred at ∼300 ns and coincides with the opening of the fingers domain. By contrast, the same transition occurs after only 22 ns in the 3HP6 simulation. Although the transitions occurred later using the Amber force field, the relative timing between the *N*-*β*-glycosyl bond rotations for the 1LV5 and 3HP6 simulations remained consistent with the Charmm27 force field.

### 3HP6 Simulations

The full polymerase fingers domain opening mechanism was characterized using the 1LV5 simulations, but the 3HP6 simulations were performed in the binary state to examine the opening process from ajar to open. As previously mentioned, in every trajectory beginning from the 3HP6 conformation the fingers domain opened quickly (always <50 ns). The general mechanism was similar to the simulations starting from the closed simulation, except faster and without the formation of the previously proposed intermediate state. The Tyr714-Glu658 hydrogen bond breaks initially, which eventually allows Tyr714 to replace the template base in the active site upon rotation of the *N*-*β*-glycosyl bond of the nucleotide that positions the polymerase in the open conformation. Once in the open conformation, the simulations persisted in that state for the remainder of each trajectory (up to 1.0 µs).

The relative rate of opening of the polymerase starting from the 3HP6 simulations suggests the potential energy barrier between the open and 3HP6 ajar conformations is relatively low compared to other barriers in the opening process. The 3HP6 ajar conformation was trapped experimentally using a double mutant and with a non-Watson-Crick base pair in the active site (dTTP-dG). Natively, the presence of a mismatch dNTP in the active site of DNA polymerase typically invokes re-opening of the fingers domain, permitting the incorrect dNTP to dissociate and allowing another dNTP to enter the active site. Considering the necessary efficiency of DNA polymerase at differentiating between correct and incorrect base pairs, it is logical to conclude the barrier between the mismatched ajar conformation (3HP6) and the original open conformation must be low so the enzyme can quickly release incorrect base pairs. This hypothesis would be consistent with our observation of a quick opening process from the 3HP6 ajar state to a conformation with the fingers domain fully open.

## Discussion

Characterization of the opening pathway for DNA polymerase is vital to fully understanding the dynamics of DNA polymerase, and providing the groundwork for future work both experimentally and computationally on the ternary protein∶DNA∶dNTP state. We performed long time-scale unbiased MD to simulate the opening of the fingers domain in *B. stearothermophilus* DNA polymerase I pre-catalysis starting from both the ajar and closed conformations in the binary state. We additionally performed simulations on the open conformation to represent the stability of the binary complex as a control experiment. Simulations were performed in triplicate using the Amber MD software with the Amber ff99SB force field and the Desmond MD software utilizing both the Amber ff99SB force field and the Charmm27 force field. All simulations, regardless of force field used or the starting conformation, progressed and reached the open conformation as expected based on experimental studies on DNA polymerase in the absence of dNTP.

We have characterized the complete opening pathway (Figure 5) based on the dynamics of each simulation beginning in the fully closed conformation. The process is initiated by the cleavage of the Tyr714-Glu658 hydrogen bond, which is followed by the formation a newly observed intermediate described by a key salt-bridge between the side chains of Arg703 in the O-helix of the fingers domain and Glu562 in the thumb domain. While the polymerase resides in this intermediate state, Tyr714 and the template base clash in the active site until the Arg703-Glu562 salt bridge breaks and the *N*-*β*-glycosyl bond of the template rotates by ∼90° allowing Tyr714 to replace the template nucleotide in the active site. These stages were observed in all three simulations starting from the 1LV5 PDB.

Further details regarding the key dihedrals in the fingers domain suggest an ordering of events in the backbone movement of the opening pathway for DNA polymerase. The Asp680φ dihedral initially rotates the N-helix downward, forcing a change in the Gly711φ and Val713ψ dihedrals that bends the O-helix while in the intermediate state. Later rotation of the same Asp680φ dihedral causes Ile716φ to rotate the O-helix enough to observe the fully open conformation by 300 ns. However, movement of these dihedrals 300–400 ns later in the simulation suggests the active site is mobile in the active site and the template DNA base can enter the active site while polymerase is in the open conformation, which was not observed crystallographically. This observation suggests that the presence of the template base in the active site may facilitate elongation via dNTP recognition.

### Impact for Future Molecular Dynamics Simulations

Although DNA polymerase I has been simulated previously using MD, no simulations have been performed using unbiased potentials over long time-scales before this study. We herein report the stability of DNA polymerase I using multiple MD packages (Desmond and Amber) utilizing two force fields (Amber ff99SB and Charmm27) on time-scales of up to 3.0 µs at the high operational temperature of a thermophilic enzyme. The simulations independently and accurately reproduced large conformational changes of DNA polymerease I known from X-ray crystallography in addition to predicting a new intermediate that would be difficult to observe experimentally. Future simulations on polymerase can be performed with confidence knowing that the current force fields have the ability to reproduce experimentally derived structures, which implies we can ask more detailed and specific questions about the polymerase dynamics not already addressed in this study.

From a broader perspective, this study also provides information about the state of our protein and nucleic acid force fields. Most MD studies perform simulations that do not reach the µs range, and only with recent technological advancements have we been able to reach these time-scales. As computational chemists simulate proteins and nucleic acids for longer time periods, we must ensure the force fields are able to maintain biologically stable structures. Most validation studies are performed on simulations of nucleic acids [30], [48] or proteins [49] separately that are typically relatively small in size, but this study allows us to evaluate the performance of these parameters simultaneously on a system (DNA polymerase I) that is over 10,000 atoms unsolvated (up to 80,000 atoms solvated). The results from the µs simulations in this study suggest the current force fields are sufficient for representing and describing the dynamics of large protein-DNA complexes on the µs time-scale. While it is unknown if these conclusions hold for even longer time-scales or larger systems, the force fields seem adequate to observe large-scale conformational changes in the current study. Future studies will focus on the mechanism of the fingers domain closing in the presence of a dNTP substrate in the active site.

## Supporting Information

Movie S1 **Movie depicting the opening process of DNA polymerase.** This movie represents the first 500 ns of the simulation began from the closed (1LV5) conformation and simulated using Desmond and the Charmm27 force field.
(MPG)
